# Supplementary material for: Relationship between exclusive breastfeeding and brain-derived neurotrophic factor in children
Source: PLoS One. 2021 Mar 4;16(3):e0248023. doi: 10.1371/journal.pone.0248023 (PMC7932083; doi:10.1371/journal.pone.0248023)
Supplement: S1 File — (DOCX) [file pone.0248023.s002.docx]

**PLEASE READ THE INSTRUCTIONS BEFORE ANSWERING THE CUESTIONS. THEN, GO ON TO QUESTION 1.**

| **BREASTFEEDING** | **BF** | 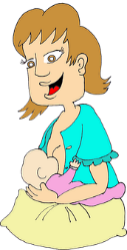 | Your child was **BREASTFED** |
| --- | --- | --- | --- |
| **FORMULA FEEDING** | **FF** | 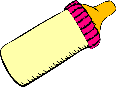 | Your child was **FORMULA-FED** |
| **COMPLEMENTARY FEEDING** | **CF** | 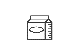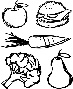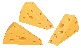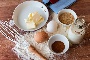 | Your child was fed with any of these solid or liquid foods: **COW MILK,** water, juices, cereals, jars, fruit, meat, fish, purée, rice, pasta, stock, eggs, or any other type of food. |

**INSTRUCTIONS**

- **QUESTION 1: MULTIPLE OPTIONS CAN BE LISTED. Put a cross in YOUR CHILD FEEDING METHOD IN EACH MONTH for the first 24 months.**
- **BF**
- **FF**
- **CF**
- **BF**
- **FF**
- **CF**
- **BF**
- **FF**
- **CF**
- **BF**
- **FF**
- **CF**
- **BF**
- **FF**
- **CF**
- **BF**
- **FF**
- **CF**
- **BF**
- **FF**
- **CF**
- **BF**
- **FF**
- **CF**
- **BF**
- **FF**
- **CF**
- **BF**
- **FF**
- **CF**
- **BF**
- **FF**
- **CF**
- **BF**
- **FF**
- **CF**

**1^st^ MO. 2^nd^ MO. 3^rd^ MO. 4^th^ MO. 5^th^ MO. 6^th^ MO. 7^th^ MO. 8^th^ MO. 9^th^ MO. 10^th^ MO. 11^th^ MO. 12^th^ MO.**

**13^th^ MO. 14^th^ MO. 15^th^ MO. 16^th^ MO. 17^th^ MO. 18^th^ MO. 19^th^ MO. 20^th^ MO. 21^st^ MO. 22^nd^ MO. 23^rd^ MO. 24^th^ MO.**

- **BF**
- **FF**
- **CF**
- **BF**
- **FF**
- **CF**
- **BF**
- **FF**
- **CF**
- **BF**
- **FF**
- **CF**
- **BF**
- **FF**
- **CF**
- **BF**
- **FF**
- **CF**
- **BF**
- **FF**
- **CF**
- **BF**
- **FF**
- **CF**
- **BF**
- **FF**
- **CF**
- **BF**
- **FF**
- **CF**
- **BF**
- **FF**
- **CF**
- **BF**
- **FF**
- **CF**
- **QUESTION 2: Was your child born at 37 OR MORE weeks of pregnancy? (37 weeks are equivalent to eight-and-a-half months)** YES NO
- **QUESTION 3: Was your child breastfed in the first hour of life?** YES NO
- **QUESTION 4: Was your child breastfed?** YES (BEGGINING month: _______; FINISHING month: _______) NO
- **QUESTION 5: Was your child formula-fed?** YES (BEGGINING month: _______; FINISHING month: ______) NO
- **QUESTION 6: At what age did you introduce complementary feeding in your child?** _______month

**ANTES DE CONTESTAR, LEA ESTAS INSTRUCCIONES Y PASE A LA PREGUNTA 1.**

| **LACTANCIA MATERNA** | **LM** | 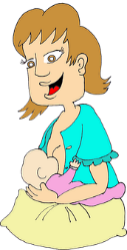 | Su hijo fue alimentado con **LECHE MATERNA** |
| --- | --- | --- | --- |
| **LACTANCIA ARTIFICIAL** | **LA** | 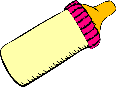 | Su hijo fue alimentado con **LECHE DE FÓRMULA o de farmacia** |
| **ALIMENTACIÓN COMPLEMENTARIA** | **AC** | 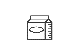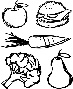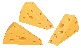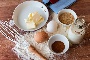 | Su hijo fue alimentado con cualquiera de los siguientes alimentos sólidos o líquidos: **LECHE DE VACA,** agua, zumos, papillas, potitos, fruta, carne, pescado, purés, arroz, pasta, caldo, huevos y otro tipo de alimentos. |

**INSTRUCCIONES**

- **PREGUNTA 1: PUEDE MARCAR VARIAS OPCIONES. Marque con una X el método de ALIMENTACIÓN de su hijo EN CADA MES durante los primeros 24 meses.**
- **LM**
- **LA**
- **AC**
- **LM**
- **LA**
- **AC**
- **LM**
- **LA**
- **AC**
- **LM**
- **LA**
- **AC**
- **LM**
- **LA**
- **AC**
- **LM**
- **LA**
- **AC**
- **LM**
- **LA**
- **AC**
- **LM**
- **LA**
- **AC**
- **LM**
- **LA**
- **AC**
- **LM**
- **LA**
- **AC**
- **LM**
- **LA**
- **AC**
- **LM**
- **LA**
- **AC**

**1º MES 2º MES 3º MES 4º MES 5º MES 6º MES 7º MES 8º MES 9º MES 10º MES 11º MES 12º MES**

**13º MES 14º MES 15º MES 16º MES 17º MES 18º MES 19º MES 20º MES 21º MES 22º MES 23º MES 24º MES**

- **LM**
- **LA**
- **AC**
- **LM**
- **LA**
- **AC**
- **LM**
- **LA**
- **AC**
- **LM**
- **LA**
- **AC**
- **LM**
- **LA**
- **AC**
- **LM**
- **LA**
- **AC**
- **LM**
- **LA**
- **AC**
- **LM**
- **LA**
- **AC**
- **LM**
- **LA**
- **AC**
- **LM**
- **LA**
- **AC**
- **LM**
- **LA**
- **AC**
- **LM**
- **LA**
- **AC**
- **PREGUNTA 2: ¿Su hijo/a nació con 37 O MÁS semanas de gestación?** (37 semanas equivale a 8 meses y medio) SÍ NO
- **PREGUNTA 3: ¿Le dio el pecho a su hijo/a en la 1ª hora de vida?** SÍ NO
- **PREGUNTA 4: ¿Le dio a su hijo/a lactancia materna?** SÍ (Mes de INICIO: _______; Mes de FINALIZACIÓN: _______) NO
- **PREGUNTA 5: ¿Le dio a su hijo/a leche de fórmula o de farmacia?** SÍ (Mes de INICIO: _______; Mes de FINALIZACIÓN: ______) NO

**PREGUNTA 6: ¿A qué edad introdujo la alimentación complementaria a su hijo?** _______mes

**Table S1. Differences between children who had information on breastfeeding and those who did not.**

|  | **Breastfeeding data (n=202)** | **No breastfeeding data (n=368)** | **p-Value** |
| --- | --- | --- | --- |
| Age (years) | 9.60 (0.69) | 9.58 (0.74) | .797 |
| Physical characteristics |  |  |  |
| *Weight (kg)* | 37.31 (9.96) | 36.28 (10.04) | .290 |
| *Height (cm)* | 141.39 (7.63) | 140.71 (7.16) | .491 |
| *BMI (kg/m^2^)* | 18.47 (3.73) | 18.14 (3.96) | .323 |
| *BF %* | 24.33 (6.46) | 23.80 (7.13) | .365 |
| *Waist circumference (cm)* | 66.40 (9.48) | 65.78 (10.38) | .487 |
| Birth weight (kg) | 3.22 (0.57) | 3.27 (0.62) | .423 |
| Mothers´ gestational age (weeks) | 38.75 (2.43) | 38.83 (2.39) | .774 |
| *BDNF (nmol/ml)* | 47.82 (9.90) | 46.55 (7.57) | .413 |
| SES |  |  |  |
| *Low* | 4 (2.2) | 4 (1.3) | .130 |
| *Medium-low* | 42 (23.0) | 52 (17.4) |  |
| *Medium* | 98 (53.6) | 151 (50.7) |  |
| *Medium-high* | 36 (19.7) | 82 (27.5) |  |
| *High* | 3 (1.6) | 9 (3.0) |  |
| Sexual maturation (Tanner stages) |  |  |  |
| *Pre-pubertal* | 68 (44.7) | 97 (44.7) | .771 |
| *Early-pubertal* | 54 (35.5) | 83 (38.2) |  |
| *Mid-pubertal* | 26 (17.1) | 34 (15.7) |  |
| *Late-pubertal* | 3 (2.0) | 3 (1.4) |  |
| *Post-pubertal* | 1 (0.7) | 0 (0.0) |  |

Data are exposed by mean ± standard deviation, except for frequency variables (SES and sexual maturation) which are shown as n (%). The values in bold indicate a statistical significance for p < 0.05, analyzed by Student’s t test (continuous variables) or Fisher´s exact test (categorical variables).

Data about participants in SES and sexual maturation variables show missing of 19 and 41%, respectively.

BMI, body mass index; BF %, body fat percentage; BDNF, brain-derived neurotrophic factor; SES, socioeconomic status.
